# Comparison of immunological and molecular methods for laboratory diagnosis of ocular toxoplasmosis in blood, serum and tears in Brazil

**DOI:** 10.1371/journal.pone.0298393

**Published:** 2024-02-06

**Authors:** Raissa Cristina Ferreira Ramos, Alynne da Silva Barbosa, Ana Luisa Quintella do Couto Aleixo, Igor Falco Arruda, Maria Regina Reis Amendoeira

**Affiliations:** 1 Laboratory of Toxoplasmosis and Other Protozooses, Oswaldo Cruz/Fiocruz Institute, Rio de Janeiro, Brazil; 2 Department of Microbiology and Parasitology, Biomedical Institute / Fluminense Federal University, Niterói, Rio de Janeiro, Brazil; 3 Laboratory of Clinical Research in Infectious Ophthalmology–Evandro Chagas National Institute of Infectology/Fiocruz, Rio de Janeiro, Brazil; National Research Center (NRC), EGYPT

## Abstract

Ocular toxoplasmosis (OT) is caused by protozoan *T*. *gondii*. Ophthalmological examination is considered the gold standard for OT diagnosis, and laboratory tests are used for diagnostic confirmation. However, these tests can present different results, which change depending on their basis, on sample type and on patients’ clinical alteration. Thus, the aim of the present study is to assess immunodiagnostic and molecular techniques applied in blood, serum and tear fluid to diagnose *T*. *gondii* infection in patients seen at an Ophthalmology Clinic. In total, 160 patients were included in the study, 40 of them had OT with active lesions (G1); 40 had OT with healed lesions (G2), 40 had non-toxoplasmic uveitis (G3) and 40 had no ocular alterations (G4). Serum samples were subjected to Immunoenzymatic Assay (ELISA) and to Indirect Immunofluorescence Reaction (IFAT) to search for anti-*T*. *gondii* IgM and IgG. Tear fluid samples were analyzed through ELISA for IgA research. All blood and tear fluid samples were subjected to conventional polymerase chain reaction (cPCR) and in a Nested PCR model for *T*. *gondii* DNA amplification with targets B1, GRA7 and REP 529. IgG and IgM anti-*T*. *gondii* was detected in serum samples from 106 and 15 patients, respectively, when combining ELISA and IFAT results. Anti-*T*.*gondii* IgA antibodies were detected in 9.2% of the tear material. Nested PCR with GRA7 target showed higher positivity in blood samples (24.4%); Nested PCR with B1 target showed a higher frequency of positivity in tears (15%). Biological samples of patients with active lesions showed the highest positivity frequencies in all immunodiagnostic assays, as well as in most PCR models. The present results highlighted the need of associating techniques with different fundamentals to confirm OT diagnosis. Furthermore, further tear fluid analyses should be performed to validate this biological material as lesser invasive alternative for the more accurate OT diagnosis.

## Introduction

Human toxoplasmosis is a parasitosis of both global distribution and zoonotic importance, because *T*. *gondii* is a facultative heteroxenic coccidia, obligate intracellular, capable of infecting homeothermic animals, in general [1]. The rates of infection caused by this protozoan are oftentimes higher in tropical environments due to their high humidity and temperatures, which favor oocysts’ sporulation in the soil [2]. Its prevalence ranges from 25% to 30%, depending on country of occurrence; these rates’ range can reach from 10% to 90% [3, 4]. This parasite’s seroprevalence in humans, in Brazil, ranges from 21.5% to 97.4% [5].

Ocular toxoplasmosis (OT) is one of the most severe clinical conditions caused by this infection. Approximately 10% of people who acquire post-natal *T*. *gondii* infection, i.e., up to 80% of children who are congenitally infected, develop ocular toxoplasmosis [6]. OT clinical manifestations result from tachyzoites’ invasion into retinal cells during the infection’s acute phase and over the chronic infection caused by the reactivation of tissue cysts found in the retina, which release bradyzoites that lead to intense inflammatory response and, consequently, to tissue destruction [7, 8]. Toxoplasmic retinochoroiditis, which may be followed by other ocular alterations capable of causing progressive visual impairment that leads to vision loss, is the most common ocular manifestation of it [7]. Among the ophthalmological surveys carried out, Brazilian cities overall have already reported the largest number of ocular toxoplasmosis cases in the world. Erichim City, Rio Grande do Sul State, Brazil, stands out within this context for accounting for the highest OT frequency in the world (17.7%), mainly because of the disease’s acquired form [9, 10]. In addition, after the outbreak that has occurred in Santa Isabel do Ivaí City, Paraná State, Brazil, OT frequency rates showed 1.73% new cases in patients with anti-*T*. *gondii* IgM antibodies [11]. In 2018, a large toxoplasmosis outbreak was reported in Santa Maria City, Rio Grande do Sul State, Brazil. In total, 29 (15.5%) of the 187 infected children had congenital toxoplasmosis and 19 children had ocular lesions, including retinochoroiditis [12].

Ophthalmological examination is considered the gold standard for OT diagnosis, and laboratory tests are used for diagnostic confirmation [13]. There are several diagnostic methods to detect toxoplasmosis, and their application depends on infections’ pathophysiological and clinical variability [14]. Diagnosis can be made through direct methods, which are techniques used to search for the parasite itself or for DNA structures often applied in blood samples, as well as through indirect techniques that aim at searching for antibodies in sera produced by the infection itself [15]. Techniques consisting of direct methods are bioassay, *T*. *gondii* isolation through cell culture and Polymerase Chain Reaction (PCR). All indirect methods consist of serological techniques [1].

Samples collected from the aqueous and vitreous humor often present more significant sensitivity results than blood samples in laboratory OT diagnosis. In addition, it is necessary adopting the Goldman-Witmer coefficient to show the intraocular production of anti-*T*. *gondii* antibodies. Furthermore, parasite’s DNA detection through the PCR technique in the aqueous and vitreous humor has shown good diagnosis’ results [1]. However, the collection of this biological sample type is extremely invasive, since it has to be performed in surgical center [16]. Toxoplasmosis laboratory diagnosis in humans can also be made through PCR. *T*. *gondii* DNA amplification through PCR application in body fluids and tissues has been used because it is efficient, as well as has relative sensitivity and specificity for this parasite’s detection [17].

Overall, OT diagnosis is challenging and mostly depends on typical clinical findings in the retina [13, 18]. In addition, laboratory examination can substantiate a presumptive clinical diagnosis in case of positive *T*. *gondii* serology or make it possible discarding it in case of negative serology [13, 18]. However, some clinical cases do not evidence clear correlation between serum antibody levels and ocular toxoplasmosis symptoms, a fact that highlights the need of further studies aimed at assessing both different laboratory techniques and biological samples to improve the diagnosis of this disease [19]. Accordingly, the aim of the present study was to assess the immunodiagnostic and molecular techniques applied to blood, serum and tear fluid to diagnose *T*. *gondii* infection in patients treated at an Ophthalmology Outpatient Clinic in Rio de Janeiro.

## Materials and methods

### Sampling, study location and participants’ awareness

Patients treated at the outpatient clinic of the Laboratory of Infectious Ophthalmology of the National Institute of Infectious Diseases (INI) Fiocruz/RJ, between April 2021 and July 2022, who agreed to participate in the study after awareness-raising, verbal invitation and signing the Informed Consent Form, were included in the study. Patients belonging to both the male and female sex, over 18 years old. These were divided into four groups: (G1) Patients with active toxoplasmic retinochoroiditis lesions, (G2) Patients with healed toxoplasmic retinochoroiditis lesions, (G3) Patients with non-toxoplasmic uveitis. In addition, individuals without ocular alterations and with negative toxoplasmosis serology (G4-control) were also sensitized to participate. Thus, 40 individuals were included in each group, and it totaled 160 patients (it formed a convenience sample).

### Ophthalmological clinical examination

Patients who accepted to participate in the study, after the invitation made by the team, underwent a clinical examination, which consisted of i) visual acuity measurement, ii) biomicroscopy, iii) fundoscopy under mydriasis and iv) applanation tonometry. Acuity measurement was performed by showing the patient objects of different sizes, at a standard distance from the eye (5m). Snellen table was used for this examination; it is a table with letters or symbols (of different sizes) organized into rows and columns. Based on the International Council of Ophthalmology classification using the Snellen chart, acuity can be set as follows: normal vision 20/12 to 20/25, mild vision loss 20/30 to 20/63, moderate loss 20 /80 to 20/160, severe loss ≤200, profound loss finger count at one meter, light perception and no light perception.

### Collection tear fluid and serum samples

Tear fluid collection from the affected eye was performed. Fluid recovery was performed with the aid of filter paper, as recommended for Schirmer Test. Paper tape was bent 0.5 cm from its end during the collection procedure; then, it was inserted in the outer third of the lower eyelid. It was left in this position until 30 mm of the tape’s height was filled with tear fluid. After collection, tear fluid from each patient was stored in sterile 1.5 mL microtubes filled with a previously prepared solution containing 300 μL sterile 1% PBS at pH = 7.4. In addition to tear fluid samples, peripheral blood samples were collected at the INI collection sector and sent to IOC/FIOCRUZ Protozoology Laboratory. Blood samples stored in tubes without anticoagulants were centrifuged at 1,000 × g, at room temperature, for 10 minutes, for total serum separation; later on, they were aliquoted in previously identified sterile microtubes, and kept at -20°C, until the time to perform the serological techniques.

### Immunological techniques for researching anti-*T*. *gondii* antibodies

Serum samples were sent to anti-*T*. *gondii* IgM and IgG antibodies research through Enzyme Immunoassay (ELISA) and Indirect Immunofluorescence Reaction (IFAT). The search for anti-*T*. *gondii* IgA in tear fluid was carried out through ELISA—samples from groups 1, 2 and 3.

ELISA kits used for anti-*T*. *gondii* IgM and IgG antibodies investigation in serum were Bioclin^®^ –Biolisa Toxoplasmosis IgG and Bioclin^®^ –Biolisa Toxoplasmosis IgM. Anti-*T*. *gondii* ELISA—Euroimmun^®^ was used to investigate IgA antibodies in tear fluid samples, based on technical recommendations by each manufacturer. IFAT was performed according to Camargo [20]. *T*. *gondii* strain RH tachyzoites, inactivated in 2% formalin, were used as antigens. Commercial anti-Human IgG (whole molecule)−FITC conjugates produced in rabbits, and anti-Human IgM (μ-chain specific)−FITC produced in goats diluted in Evans Blue solution, both provided by Sigma-Aldrich^®^ diluted in Evans Blue solution, were adopted to detect anti-*T*. *gondii* antibodies. Samples were considered positive when total fluorescence was observed on tachyzoite’s surface at dilution equal to, or higher than, 1:16.

### Molecular *T*. *gondii* Diagnosis

DNA extraction from blood and tear fluid samples was performed by using a Purelink Genomic DNA Mini kit (Invitrogen^®^), according to manufacturer’s recommendations. All collected samples were also first subjected to PCR assays based on using primers GAPDH-F 5’-CCTTCATTGACCTCAACTACAT-3’; GAPDH-R: 5’- CCAAAGTTGTCATGGATGACC -3’ [21], which amplify the DNA fragment of the mammalian glyceraldehyde 3 phosphate dehydrogenase protein gene; it was done to assess whether DNA extraction was properly performed.

Successfully extracted samples were subjected to *T*. *gondii* DNA research in blood and tear fluid by means of the amplification of the DNA fragment of the B1 genes [22, 23], as well as repetitive regions 529 bp (REP 529) [24–27] and GRA7 [28]. Reactions were performed through the conventional one-phase PCR model (cPCR), and in the two-phase Nested PCR model. Primers used in each PCR, cycles used in thermal cycler and the expected size of the band visualized in agarose gel are described in S1 Table.

### Statistical analyses

All general data including sex, age, patient’s ophthalmological examination data, and laboratory data were entered into Microsoft Excel tables. Results from the applied techniques were descriptively analyzed and statistically compared to assess agreement between them, after all collection and laboratory procedures were carried out. Cohen’s Kappa concordance index (IK) was used for this comparison; it was interpreted according to Landis and Koch [29]; when < 0, there is no agreement; between 0 and 0.20, there is minimal agreement; between 0.21 and 0.40, there is fair agreement; between 0.41 and 0.60, there is moderate agreement; between 0.61 and 0.80, there is substantial agreement; between 0.81 and 1.0, there is almost perfect agreement. In addition, McNemar’s test and Fisher’s exact test were also used. McNemar’s test was interpreted based on the null hypothesis, i.e., assumingly, laboratory techniques agree in this protozoan’s investigation: p value was higher than 0.05. Comparisons that recorded p≤0.05 in the Fisher’s Exact test were statistically significant. All these analyses were performed in QuickCalcs Graph Pad^®^ software. Furthermore, sensitivity, specificity, positive (PPV) and negative (NPV) predictive values were also calculated between ELISA and IFAT, in serum samples, as well as Nested PCR models for the B1, GRA7 and REP 529 gene targets in blood and tear fluid. Techniques that have presented the highest frequencies in *T*. *gondii* diagnosis results were used as gold standard.

### Ethical considerations

The present study was approved by the Human Research Ethics Committee of Oswaldo Cruz Institute/Fiocruz, under CAE number 39200820.1.0000.5248 and opinion n. 4.377.191, and by the Research Ethics Committee of Evandro Chagas National Institute of Infectious Diseases—INI/Fiocruz under CAE number 39200820.1.3001.5262 and opinion n. 4,425,977.

## Results

In total, 89 (56.6%) of all treated patients belonged to the female sex and 71 (44.4%) patients belonged to the male sex. Research participants were in the age group 19–83 years (mean age: 44 ± 14) and had already undergone more than one ophthalmological clinical care at the Outpatient Clinic (S2 Table). Regarding visual acuity, 36 (22.5%) patients had some alteration in the right eye, only; and 31 (19.4%) patients only had it in the left eye. In addition, 41 (52.6%) patients had alterations in both eyes and 52 (32.5%) patients had no ocular alteration, at all.

As for clinical cases with unilateral retinochoroiditis, 59% of patients belonged to G1 and 41% of them belonged to G2. Bilateral retinochoroiditis was seen in 21.1% of patients in G1, whereas 78.9% patients in G2 had it. Regarding lesion location, most patients with central lesions were in G2 (54.1%) and those with peripheral lesions were in G1 (62.2%). Only six clinical cases in G2 showed evidence of both peripheral and central lesions. Most patients in groups 1 (48.2%) and 2 (51.8%) had more than two lesions. Furthermore, most patients with alterations in G1 and G2 recorded lesions measuring from 1 to 2 DD (Disc Diameter) (S2 Table).

Anti-*T*. *gondii* antibodies of the IgG and IgM classes were detected in serum samples from 106 (66.2%) and 15 (9.4%) patients, respectively when associating the results of the ELISA with the IFAT. All patients in G1 and G2, in addition to 26 patients in G3, were seropositive for class G antibodies. Anti-*T*. *gondii* IgM antibodies were detected in 8 (20%) patients in G1, in 5 (12.5%) patients in G2 and in 2 (5%) patients in G3 (Data not shown in table). Overall, anti-*T*. *gondii* immunoglobulins investigated in serum were more often detected by ELISA than by IFAT, except for IgM, in G3. All patients positive for IgG detected through IFAT were also positive through ELISA detection, except for one sample from a patient in G3. All samples with IgM anti-*T*.*gondii* detected through IFAT in G1 were also seroreactive through ELISA testing. However, only 2 patients in G2 were positive through ELISA and seroreactive through IFAT. IFAT was capable of recovering one more seroreactive sample in G3 than ELISA in IgM research (Table 1). Comparisons between IFAT and ELISA serological methods for IgG and IgM detection presented substantial and moderate classification, respectively these findings were corroborated by McNemar Test and Fisher’s Exact Test (Table 2).

**Table 1:** Frequency of patients with anti-*Toxoplasma gondii* IgM and IgG antibodies detected through ELISA and IFAT applied to serum, as well as with positive PCR in blood assessed for gene targets B1, GRA7 and REP 529.

Techniques	G1 (n = 40)	G2 (n = 40)	G3 (n = 40)	G4 (n = 40)	Total (n = 160)
ELISA IgM	8 (20%)	4 (10%)	1 (2.5%)	0 (0%)	13 (8.1%)
ELISA IgG	40 (100%)	40 (100%)	25 (62.5%)	0 (0%)	105 (65.6%)
IFAT IgM	3 (7.5%)	3 (7.5%)	2 (5%)	0 (0%)	8 (5%)
IFAT IgG	40 (100%)	39 (97.5%)	20 (50%)	0 (0%)	99 (61.9%)
cPCR B1	10 (25%)	1 (2.5%)	3 (7.5%)	0 (0%)	14 (8.7%)
PCR nested B1	19 (47.5%)	13 (32.5%)	6 (15%)	0 (0%)	38 (23.7%)
cPCR 529	1 (2.5%)	6 (15%)	0 (0%)	0 (0%)	7 (4.4%)
PCRnested 529	16 (40%)	14 (35%)	7 (17.5%)	0 (0%)	37 (23.1%)
cPCR GRA7	1 (2.5%)	0 (0%)	0 (0%)	0 (0%)	1 (0.6%)
PCRnested GRA7	18 (45%)	14 (35%)	7 (17.5%)	0 (0%)	39 (24.4%)

G1: Patient with active ocular toxoplasmosis; G2: patient with healed ocular toxoplasmosis; G3: patient with non-toxoplasmic uveitis; G4: patient without ocular injury; n: absolute number; %: frequency; cPCR: Polymerase chain reaction performed through only one reaction step; Nested PCR: Polymerase chain reaction performed through two reaction Steps.

**Table 2:** Comparison between immunodiagnostic tests in serum samples, according to the Kappa index, McNemar and Fisher’s Exact tests, to detect IgG and IgM antibodies.

Serological techniques	N = (120)	Kappa	McNemar	Fisher’s Exact
ELISA IgM serum versus IFAT IgM serum	6 (5%)	0.533	0.1824	<0.0001*
ELISA IgG serum versus IFAT IgG serum	98 (81.6%)	0.741	0.0771	<0.0001*

Kappa < 0, there is no agreement; between 0 and 0.20, there is minimum agreement; between 0.21 and 0.40, there is reasonable agreement; between 0.41 and 0.60, there is moderate agreement; between 0.61 and 0.80, there is substantial agreement; and between 0.81 and 1.0, there is almost perfect agreement

*Statistically significant.

Of the 160 samples collected, it was possible detecting *T*. *gondii* DNA in blood samples from 53 (33.1%) patients: 25 (15.6%) from G1, 18 (11.2%) from G2 and 10 (6.2%) from G3, by associating the results recorded for all PCR models with different assessed targets. The Nested PCR model showed a higher amplification frequency than cPCR for all targets used (Table 1).

GRA7 Nested PCR (24.4%) showed the highest positivity frequency; it was followed by both the B1 gene Nested PCR (23.7%) and the REP 529 Nested PCR (23.1%). The highest frequency of positive samples in the Nested PCR model was recorded for G1; it was followed by G2 and G3 (Table 1). All positive samples in cPCR were also positive in Nested PCR. Patients who did not have eye lesion, included in G4, had their negative *T*. *gondii* result confirmed through serological and molecular techniques (Table 1).

The search for anti-*T*. *gondii* IgA antibodies in tear fluid samples showed 9.2% (11/120) positivity frequency through ELISA. They were more often detected in samples from patients in G1 (20%), who were followed by those in G3 (5%) and G2 (2.5%) (Table 3).

**Table 3:** Frequency of patients with anti-*Toxoplasma gondii* IgA antibodies detected through ELISA in tear fluid samples and with positive PCR in tear fluid with gene targets B1, GRA7 and REP 529.

Techniques	G1 (n = 40)	G2 (n = 40)	G3 (n = 40)	G4 (n = 40)		Total (n = 160)
ELISA IgA	8 (20%)	1 (2.5%)	2 (5%)	*		11 (9.2%) **
cPCR B1	10 (25%)	4 (10%)	1 (2.5%)	0 (0%)		15 (9.3%)
PCR nested B1	17 (42.5%)	5(12.5%)	2 (5%)		0 (0%)	24 (15%)
cPCR 529	1 (2.5%)	0 (0%)	0 (0%)	0 (0%)		1 (0.6%)
PCRnested 529	7 (17.5%)	3 (7.5%)	1 (2.5%)	0 (0%)		11 (6.9%)
cPCR GRA7	0 (0%)	0 (0%)	0 (0%)	0 (0%)		0 (0%)
PCRnested GRA7	8 (20%)	3 (7.5%)	0 (0%)	0 (0%)		11 (6.9%)

G1: Patient with active ocular toxoplasmosis; G2: patient with healed ocular toxoplasmosis; G3: patient with non-toxoplasmic uveitis; G4: patient without ocular injury; n: absolute number; %: frequency; cPCR: Polymerase chain reaction performed at only one reaction step; Nested PCR: Polymerase chain reaction performed at two reaction steps

*: ELISA IgA was not performed in G4

**: Rate calculation was based on 120 patients (G1, G2 and G3).

Of the 160 samples collected, it was possible detecting *T*. *gondii* DNA in tear fluid samples from 35 (21.9%) patients, of whom 23 (14.4%) were in G1, 10 (6.2%) were in G2 and 2 (1.2%) were in G3, by associating results recorded for all PCR models with the different assessed targets. The highest frequencies of positive samples in tear fluid in PCR were recorded for the Nested PCR model, based on amplification of the B1 gene fragment; it was followed by cPCR (with the same target), and by Nested PCR models, with the targets GRA7 and REP 529 bp. Notably, tear fluid samples from group 1 were the ones presenting the highest DNA amplification rates in all PCR models included in the present study, at the three herein used targets (Table 3).

Overall, greater positivity was observed in molecular *T*. *gondii* diagnosis carried out through the Nested PCR model, with immunodiagnosis for all anti-*T*. *gondii* antibody classes investigated in the present study. There was higher copositivity in G1, between serological diagnosis anti-*T*. *gondii* diagnosis through the Nested PCR B1 model by breaking down this correlation based on groups of patients included in the study. It was followed by Nested GRA7 and Nested REP 529. This correlation was more evident between serological diagnosis and Nested PCR REP 529 in G2; it was followed by Nested GRA7 and Nested B1. Similar copositivity frequencies were observed between IgA anti-*T*. *gondii* diagnosis and Nested PCR models, applied to tear fluids, both in G1 and G2. However, only three biological samples from the same patients showed simultaneous copositivity between IgA ELISA and the three herein adopted Nested PCR models: two patients in G1 and one patient in G2. Copositivity was observed between serum IgM and tear IgA detection through the Nested PCR models REP 529 and GRA7, in G3 (Table 4).

**Table 4:** Copositivity between molecular techniques for *T*. *gondii* DNA amplification in blood with gene targets B1, GRA7 and REP 529 bp, through conventional PCR and Nested models associated with immunodiagnosis to research different immunoglobulins classes in serum and fluid tear.

	Immunodiagnosis	cPCR B1	Nested PCR B1	cPCR 529	Nested PCR 529	cPCR GRA7	Nested PCR GRA7
	Serum ELISA IgM	2 (5%)	5 (12.5%)	1 (2.5%)	4 (10%)	0	4 (10%)
G1 (n = 40)	Serum ELISA IgG	10 (25%)	19 (47.5%)	1 (2.5%)	16 (40%)	1 (2.5%)	18 (45%)
	Tear fluid ELISA IgA	1 (2.5%)	4 (10%)	1 (2.5%)	4 (10%)	1 (2.5%)	4 (10%)
	Serum ELISA IgM	0	3 (7.5%)	2 (5%)	4 (10%)	0	3 (7.5%)
G2 (n = 40)	Serum ELISA IgG	1 (2.5%)	13 (32.5%)	6 (15%)	14 (35%)	0	14 (35%)
	Tear fluid ELISA IgA	0	1 (2.5%)	1 (2.5%)	1 (2.5%)	0	1 (2.5%)
	Serum ELISA IgM	0	0	0	1 (2.5%)	0	1 (2.5%)
G3 (n = 40)	Serum ELISA IgG	3 (7.5%)	6 (15%)	0	7 (17.5%)	0	7 (17.5%)
	Tear fluid ELISA IgA	0 (0%)	0	0	1 (2.5%)	0	1 (2.5%)
	Serum ELISA IgM	2 (16%)	8 (6.6%)	3 (2.5%)	9 (7.5%)	0	8 (6.6%)
Total (n = 120)	Serum ELISA IgG	14 (11.6%)	38 (31.6%)	7 (5.8%)	37 (30.8%)	1 (0.8%)	39 (32.5%)
	Tear fluid ELISA IgA	1 (0.8%)	5 (4.1%)	2 (16%)	6 (5%)	1 (0.8%)	6 (5%)

Substantial agreements were observed between the Nested PCR models REP 529 and Nested PCR GRA7, Nested PCR B1 and Nested PCR GRA7, which was followed by a moderate agreement between Nested PCR B1 and Nested PCR REP 529, in the comparison of molecular techniques applied to detect *T*. *gondii* DNA in blood samples by using the Cohen’s Kappa index. These substantial and moderate agreements were confirmed through McNemar’s test, which were also statistically significant in Fisher’s Exact Test (p≤0.05). Substantial agreement was also identified between cPCR and Nested PCR, with the B1 gene, for protozoan DNA diagnosis in patients’ tear fluid. Furthermore, a moderate agreement was recorded based on the comparison of results generated between Nested PCR models REP 529 and Nested PCR GRA7, even in tear fluids. Unlike what was observed in comparisons made between molecular techniques in blood samples and in tear fluids, the comparison classified as substantial, according to Landis and Koch [29], was not corroborated through McNemar Test, because p value was lower than 0.05 (Table 5).

**Table 5:** Comparison of molecular tests applied to detect *Toxoplasma gondii* DNA in blood and tear fluid samples, based on Kappa index, through McNemar and Fisher’s Exact tests, with gene targets B1, GRA7 and REP 529.

**Molecular techniques (Blood)**	**Number (n = 160)**	**Kappa**	**McNemar**	**Fisher’s Exact**
cPCR B1 versus Nested PCR B1	14 (8.7%)	0.471	<0.0001	<0.0001*
cPCR B1 versus cPCR 529	0	0.062	0.1904	1
cPCR B1 versus Nested PCR 529	9 (5.6%)	0.259	<0.0001	0.0007*
cPCR B1 versus cPCR GRA7	0	0.012	0.0019	1
cPCR B1 versus Nested PCR GRA7	8 (5%)	0.199	<0.0001	0.0062*
Nested PCR B1 versus cPCR 529	5 (3.1%)	0.16	<0.0001	0.0087
Nested PCR B1 versus Nested PCR 529	24 (15%)	0.53	1	<0.0001*
Nested PCR B1 versus cPCR GRA7	1 (0.6%)	0.04	<0.0001	0.2753
Nested PCR B1 versus Nested PCR GRA7	28 (17.5%)	0.641	1	<0.0001*
cPCR 529 versus Nested PCR 529	7 (4.3%)	0.264	<0.0001	<0.0001*
cPCR 529 versus cPCR GRA7	0	0.011	<0.0001	1
cPCR 529 versus Nested PCR GRA7	4 (2.5%)	0.108	<0.0001	0.0606
Nested PCR 529 versus cPCR GRA7	0	0.012	<0.0001	1
Nested PCR 529 versus Nested PCR GRA7	29 (18.1%)	0.689	0.8137	<0.0001*
cPCR GRA7 versus Nested PCR GRA7	1 (0.6%)	0.038	<0.0001	0.2438
**Molecular techniques (Tear fluid)**	**Number (n = 160)**	**Kappa**	**McNemar**	**Fisher’s Exact**
cPCR B1 versus Nested PCR B1	15 (9.3%)	0.739	0.0077	<0.0001*
cPCR B1 versus cPCR 529	1 (0.6%)	0.115	0.0005	0.0938
cPCR B1 versus Nested PCR 529	3 (1.8%)	0.164	0.5023	0.0698
cPCR B1 versus cPCR GRA7	0	0	0.0003	1
cPCR B1 versus Nested PCR GRA7	2 (1.2%)	0.081	0.5224	0.275
Nested PCR B1 versus cPCR 529	1 (0.6%)	0.069	<0.0001	0.15
Nested PCR B1 versus Nested PCR 529	5 (3.1%)	0.211	0.1175	0.0125
Nested PCR B1 versus cPCR GRA7	0	0	<0.0001	1
Nested PCR B1 versus Nested PCR GRA7	3 (1.8%)	0.085	0.0259	0.2161
cPCR 529 versus Nested PCR 529	1 (0.6%)	0.157	0.0133	0.0688
cPCR 529 versus cPCR GRA7	0	0	1	1
cPCR 529 versus Nested PCR GRA7	1 (0.6%)	0.157	0.0044	0.0688
Nested PCR 529 versus cPCR GRA7	0	0	0.0026	1
Nested PCR 529 versus Nested PCR GRA7	5 (3.1%)	0.414	0.7728	0.0002*
cPCR GRA7 versus Nested PCR GRA7	0	0	0.0026	1

Kappa < 0, there is no agreement; between 0 and 0.20, there is minimum agreement; between 0.21 and 0.40, there is reasonable agreement; between 0.41 and 0.60, there is moderate agreement; between 0.61 and 0.80, there is substantial agreement; and between 0.81 and 1.0, there is almost perfect agreement

*Statistically significant

Comparisons of results recorded for the immunodiagnostic tests applied to detect immunoglobulins in different biological samples were carried out only in groups other than G4 to confirm the negative serological and molecular result recorded for this group, i.e., patients with clinically diagnosed ocular lesions. Higher copositivity frequency between immunodiagnostic methods for anti-*T*. *gondii* serum and IgA in tear fluid was recoded for G1. Only one patient from group 2 and one from G3 showed copositivity among different antibody classes; G2 was copositive for IgM and IgG sera, through IFAT and ELISA, and for IgA in tear fluid. However, this positive correlation was observed based on the detection of IgG through ELISA and IFAT, and IgA in the tear fluid, in G3 (Table 6).

**Table 6:** Copositivity between immunodiagnostic techniques to research different immunoglobulin classes among serum and tear fluid samples.

	Techniques	Serum ELISA IgM	Serum IFAT IgM	Serum ELISA IgG	Serum IFAT IgG	Total (n = 120)
**G1 (n = 40)**	tear fluid ELISA IgA	4 (10%)	3 (7.5%)	8 (20%)	8 (20%)	8 (6.6%)
**G2 (n = 40)**	tear fluid ELISA IgA	1 (2.5%)	1 (2.5%)	1 (2.5%)	1 (2.5%)	1 (0.8%)
**G3 (n = 40)**	tear fluid ELISA IgA	0	0	1 (2.5%)	1 (2.5%)	1 (0.8%)

Classifications between agreements between techniques ranged from minimal to reasonable based on the comparison of IgA detection frequency in tear fluid versus the detection of serum antibodies (Table 7).

**Table 7:** Comparison between immunodiagnostic tests in serum and tear fluid samples based on Kappa index, and McNemar and Fisher’s Exact tests for IgG, IgM and IgA antibodies.

Serological techniques	N = (120)	Kappa	McNemar	Fisher’s Exact
Tear ELISA IgA versus serum ELISA IgM	5 (4.2%)	0.352	0.7893	0.0022*
Tear ELISA IgA versus serum IFAT IgM	4 (3.3%)	0.373	0.5465	0.0023*
Tear ELISA IgA versus serum ELISA IgG	10 (8.3%)	0.008	<0.0001	1
Tear ELISA IgA versus serum IFAT IgG	10 (8.3%)	0.02	<0.0001	0.6868

Kappa < 0: there is no agreement; between 0 and 0.20, there is minimum agreement; between 0.21 and 0.40, there is reasonable agreement; between 0.41 and 0.60, there is moderate agreement; between 0.61 and 0.80, there is substantial agreement; and between 0.81 and 1.0, there is almost perfect agreement

*Statistically significant.

Sensitivity, specificity, positive (PPV) and negative (NPV) predictive values were calculated based on the indirect or direct diagnostic method, by using the highest positivity rate. The highest sensitivity value among the serological methods was recorded through ELISA applied to IgG. Furthermore, specificity values found through IFAT for both IgM and IgG were higher than the other ones. As for methods used in blood samples, Nested PCR model for GRA7 target was the one presenting the highest sensitivity, specificity, PPV and NPV values. With respect to tear fluid, Nested PCR applied to amplify the DNA fragment of the B1 gene was the one showing the highest sensitivity and NPV values. However, Nested PCR application to amplify *T*. *gondii* DNA from REP 529 was the one accounting for the highest specificity and PPV values (Table 8).

**Table 8:** Sensitivity, specificity, positive (PPV) and negative (NPV) predictive value results between ELISA and IFAT applied to serum samples, as well as Nested PCR models for B1, GRA7 and REP 529 gene targets in blood and tear fluid samples.

Technique	Sensitivity	Specificity	PPV	NPV
ELISA IgM^1^	75%	95%	46%	98%
ELISA IgG^1^	98%	88%	93%	98%
IFAT IgM^2^	46%	98%	75%	95%
IFAT IgG^2^	93%	98%	98%	88%
Nested PCR B1 (blood)^3^	73%	90%	71%	91%
Nested PCR 529 (blood)^3^	71%	91%	73%	90%
Nested PCR GRA7 (blood)^4^	74%	93%	78%	91%
Nested PCR B1 (tear fluid)^3^	27%	85%	12%	94%
Nested PCR 529 (tear fluid)^4^	20%	95%	45%	87%
Nested PCR GRA7 (tear fluid)^4^	12%	94%	27%	85%

1: IFAT used as gold standard method (PO); 2: ELISA used PO method; 3. Nested PCR GRA7 used as PO method; 4: Nested PCR B1 used as PO method.

## Discussion

Infection caused by *T*. *gondii* can be indirectly and directly detected in both blood and tear fluid samples, in different groups of patients, regardless of their clinical outcome, by different laboratory techniques used to diagnose infection caused by this protozoan in patients with and without OT. Overall, most patients clinically diagnosed with OT showed unilateral alterations in compliance with descriptions made for individuals from different Brazilian states [30–35].

ELISA showed slightly higher positivity than IFAT in anti-*T*. *gondii* IgG antibodies’ detection. Greater positivity in ELISA than in IFAT was also evidenced in serum samples from different patients in Rio de Janeiro City, RJ, and in Oriximiná City, Pará State in the search for anti-*T*. *gondii* IgG [36, 37], as well as in vitreous humor samples of patients with OT [7]. There was also agreement in IgM diagnosis between the serological techniques. However, this finding was classified as moderate, since most results were obtained through ELISA in samples from groups 1 and 2. Low agreement between serological techniques to detect IgM was also reported in a previous study carried out in Rio de Janeiro city, since the researchers also showed greater positivity through ELISA than through IFAT [36].

Lack of complete agreement between IFAT and ELISA was already expected, since antibodies detected through these serological techniques react to different structures of the herein assessed protozoan. IFAT detects antibodies only produced against the parasite’s surface antigens. ELISA, in its turn, detects antibodies produced against cytosolic, metabolic and surface antigens [36, 37]. Thus, although the detection of IgG presents substantial agreement, it is still not perfect, and requires the use of different associated serological techniques for the diagnosis of parasites. This finding becomes even more important when it comes to IgM detection [38]. The largest sample of seroreactive anti-*T*. *gondii* individuals was expected to be found in groups 1 and 2, because these patients were previously clinically diagnosed with OT—this finding highlights the IgM positivity in group 1. It is important emphasizing that the parasites may be intensely reproducing in patients with this lesion and stimulating the beginning of a humoral adaptive immune response, whose first immunoglobulins to be excreted are those belonging to class IgM. However, IgM diagnosis must be analyzed with caution in serum samples of patients in groups 1, 2 and 3, since it may correspond to residual IgM persisting in individuals’ body for approximately 12 months, even at low titers [38–40].

All G3 seropositive patients were simultaneously positive for IgM and IgG. The detection of both immunoglobulins has also been reported in patients with ocular changes not associated with *T*. *gondii*, in São Paulo [8]. Anti-*T*. *gondii* antibodies’ occurrence in this group of patients may be indicative of asymptomatic infection caused by this protozoan, since these patients were clinically diagnosed with non-toxoplasmic infectious uveitis. Nevertheless, the possibility of the joint action taken by this protozoan along with another etiological agent in patients’ clinical ocular alteration cannot be ruled out.

Although serological techniques are widely used in the laboratory environment to diagnose toxoplasmosis, their results must be analyzed with caution, because it is known that the detection of serum antibodies in ocular toxoplasmosis is not directly correlated to the observed clinical changes [19]. One of the limiting factors for serology lies on this parasitosis’ prevalence in the investigated population, which is high in Brazil [5]. This epidemiological scenario ends up increasing the frequency of seroreactive individuals who remain in this condition throughout their lives, without being associated with ocular alterations caused by the herein assessed protozoan. Thus, more sensitive laboratory techniques such as the polymerase chain reaction should be increasingly used and validated for OT diagnosis.

Overall, with all the molecular targets applied in PCR in the current study, Nested PCR models were the ones presenting the highest frequencies of positive results in blood samples. Similar results about the best Nested PCR performance in comparison to cPCR for molecular *T*. *gondii* diagnosis have been reported in other studies [8, 23, 27, 41]. The best Nested PCR performance to detect *T*. *gondii* DNA is associated with greater sensitivity and specificity in comparison to PCR performed in one step. Because the sample is used in the second PCR, which actually consists of a previously amplified target DNA fragment, primers are used in the second reaction that amplifies internal and smaller DNA fragments. These results highlight the importance of this molecular diagnostic model to detect parasitism by *T*. *gondii* in biological samples that may present little parasitic material for diagnosis purposes.

Nested PCR reactions to the GRA7 target were the ones showing the largest numbers of *T*. *gondii* DNA amplifications in blood samples; they were followed by reactions to B1 and REP 529 bp. Comparisons of these B1 and REP 529 bp molecular targets to GRA7 showed the highest agreement scores. In addition, the GRA7 target showed the highest sensitivity, specificity and predictive value levels in molecular diagnosis of this protozoan. Comparisons to the literature regarding GRA7 primers application in Nested PCR to detect *T*. *gondii* DNA fragments were impaired, since no articles were found in the literature that used this molecular marker to detect *T*. *gondii* in biological samples; the current study is the first to assess the performance of this marker in material collected from OT patients. The choice for this target as diagnostic tool for *T*. *gondii* was based on results recorded *in vitro*, when primers that amplify the GRA7 DNA fragment were designed and assessed [28]. Among advantages of GRA7 as molecular target to diagnose this parasite, the following ones stands out: the existence of several nucleotide sequences deposited in public banks of both clonal and atypical strains of the protozoan that generally occur in Brazil; and the mandatory expression of this gene in the parasitophorous vacuole formation, which is essential for intracellular infections caused by all infective protozoan forms and, mainly, due to its high conservation in the parasite genome, overtime [28].

Although group 1 presented the best results among the other ones, sensitivity shown through molecular techniques can be considered low because they regard patients with active lesion. It should be noticed that these patients were already receiving eye care; six of them were already undergoing drug treatment. This conduct may have interfered with this protozoan’s molecular diagnosis, because the used drugs are efficient in ruling out free tachyzoite and bradyzoite forms. Furthermore, patients included in the study were at different infection–evolution times, and this situation may also have interfered with the evidenced sensitivity.

IgA was mainly detected in tear fluid of patients clinically diagnosed with OT, with emphasis on G1, in the present study. The strong association between active ocular disease and tear IgA production was also observed in OT patients, in Brazil and Egypt [16, 42]. Overall, definitive OT diagnosis occurs based on the parasite’s direct or indirect detection in the aqueous humor; this fluid is considered to be the best protozoan-detection source [6, 43]. However, these fluids’ collection is invasive and needs to be performed in Surgical Center. Thus, tear fluid has been evaluated by different authors with lesser invasive sampling alternative to detect this immunoglobulin [16, 42, 44–46].

IgA detection in the tear fluid of G2 and G3 patients may be associated with the presence of protozoan cysts in ocular tissues, or even with cysts found in other locations. IgA detection in tear fluid of patients with healed OT, as evidenced in G2, has also been reported in patients in the Netherlands, Egypt and Brazil [42, 44, 46]. Firstly, stimuli from cysts found in the eyeball or in other tissues can lead to persistent *T*. *gondii* antigens’ presentation through the rupture of these cysts, without clinical manifestations in patients [47]. This process would result in detectable serum IgA levels that are kept in the mucosa by circulating lymphocytes. Another possibility lies on the fact that the chronic stimulation of intestinal mucosa may occur due to continuous exposure to *T*. *gondii*, mainly in places with high parasite prevalence, as in Brazil [46]. sIgA detection is often quite controversial in indirect *T*. *gondii* diagnosis, because the literature only provides few information on this immunoglobulin’s kinetics in patients with and without OT. Some authors have already reported the occurrence of sIgA in serial samples collected for six years in patients with healed OT; this finding indicates likely residual IgA [48]. This fact could not be discarded at the time to diagnose patients in G2 and G3, in the current study.

Concordance between IgA diagnosis in tear fluid and serum immunoglobulins after applying the Cohen’s Kappa test was classified as minimal to fair. Low concordances classified as minimal were observed between IgA diagnostic comparisons in tear fluid and serum IgG. This finding was already expected, since serum IgG diagnosis ends up having little relevance in OT diagnosis, mainly in populations with high seroprevalence for *T*. *gondii* [16]. Thus, the tears of many individuals, who were chronically infected or not, contain IgA antibodies against *T*. *gondii;* however, it is not known whether the observed antibody responses result from common mucosal immune responses against *T*. *gondii*, or whether they represent the natural repertoire of antibodies [44]. This last assumption, if proven, could explain IgA presence in tear fluid of G3 patients.

Nested PCR model with the B1 target showed the highest frequency of positive samples in tear fluid; it amplified the parasite’s DNA fragment. The greatest sensitivity evidenced in Nested with B1 gene, in comparison to other models and targets, seems to be directly related to the smaller size of the final amplified DNA fragment that was generated by this reaction. Similar results were reported to detect the same *T*. *gondii* DNA fragment in amniotic fluid samples of pregnant women, in São Paulo [23]. Thus, it is recommended using primers that amplify small DNA fragments in biological samples that may have a smaller number of parasites due to infection time and the chosen treatment. Specificity indices and positive predictive value recorded for Nested with B1 gene were below the number of DNA amplifications generated with the other molecular targets, also in the Nested PCR model, although it led to the highest sensitivity indices and negative predictive values. Nested PCR with B1 did not diagnose all positive samples detected through the other molecular targets. This result was corroborated by the low concordance evidenced by comparisons between this Nested PCR model and the other ones.

Molecular *T*. *gondii* diagnosis was mainly observed in the tear fluid of individuals with active ocular lesions (G1). This result was already expected, given the physiology of the herein assessed parasite in primary infection or in recurrence cases. Molecular diagnosis was also assessed in fluids of G2 patients; however, it was lesser frequent when the lesion healed, and the infection was chronic. Surprisingly, DNA bands were seen on electrophoresis compatible with *T*. *gondii* DNA fragment amplification in three samples of individuals with non-toxoplasmic uveitis. It should be noticed that, among the three patients in this condition, two were serologically positive for IgM through IFAT and for IgG through IFAT and ELISA. Accordingly, results highlighted the importance and applicability of molecular diagnosis in a sample of tear fluid to confirm *T*. *gondii*, since tears are the secretion accounting for lubricating and protecting the site from likely infection, and for leading to a more specific OT diagnosis. According to the reference literature, no studies based on this methodological approach were retrieved; thus, the present research is the first to analyze tear fluid through molecular analysis to diagnose *T*. *gondii*.

None of the herein applied molecular techniques was able to detect all confirmed OT cases, since 6 G1 patients and 17 G2 ones were negative based on all conventional and nested PCRs, both performed in blood and tear fluid samples. Therefore, the use of a commercial master mix may have interfered with the performance of molecular diagnostic methods, since reagent concentrations are already defined in advance, and they are not always adequate to homogeneously amplify different protozoan DNA fragments in different amounts and types of biological samples. However, using the commercial mix optimized the laboratory routine, mainly when it comes to Nested PCR, which ended up taking longer to be performed. Thus, it is necessary standardizing the molecular methods used in the present study by taking into consideration the molecular target to be amplified, as well as the reagent concentrations used for its detection.

The size of ocular lesions observed in G1 patients may have given negative contribution to the non-detection of parasitic DNA in biological samples of patients in this group. It is known that PCR, alone, is more sensitive to detect *T*. *gondii* DNA in samples of patients who have ocular lesions equal to, or bigger than, 3 DD (Disc Diameter) [38]. This size was little evidenced in patients in the current study, since most of them, including the six negative patients in all PCRs, had lesions ranging from 1 to 2 DD.

## Conclusion

Laboratory techniques association with different fundamentals used in different biological samples, in the present study, has valued the recorded result since it minimized likely generated losses. Immunodiagnosis of both serum immunoglobulins, with emphasis on IgM and IgA, detected in tear fluid, showed the highest diagnostic frequencies in active OT cases. However, it was not possible to indirectly identify immunoglobulins in biological samples of all patients included in this group, since patients are not always at the acute infection phase, and this condition may point out low immunodiagnosis performance with these markers. Overall, Nested PCR presented the highest diagnostic of *T*. *gondii* DNA frequency, both in blood and tear fluid samples; it should, therefore, be used when PCR with electrophoresis is the only option for molecular testing. Accordingly, the molecular target GRA7 in blood samples showed high performance and it can be applied in routine laboratory diagnosis. However, the B1 gene in tear fluid was the one presenting the best result, since it amplified the parasite’s DNA, mainly in fluids of patients with OT. In light of the foregoing, further analyses must be applied to tear fluid used as alternative sample to diagnose OT, including other small-sized molecular targets and more sensitive diagnostic techniques, to highlight real-time PCR and digital PCR.

## Supporting information

S1 Table Methodological information on polymerase chain reactions used to diagnose *Toxoplasma gondii* in blood and tear fluid samples of patients with suspected ocular toxoplasmosis. (DOCX)

S2 Table General and clinical features of patients seen between April 2021 and July 2022, at the Outpatient Clinic of the Ophthalmology Laboratory of the National Institute of Infectious Diseases of Fiocruz. *G1: Patient with active ocular toxoplasmosis; G2: patient with healed ocular toxoplasmosis; G3: patient with non-toxoplasmic uveitis; G4: patient without ocular injury. RE: right eye; LE: Left eye. F: Female sex; M: Male sex. DD: Disc Diameter.
(DOCX)
