# Supplementary material for: Comparison of immunological and molecular methods for laboratory diagnosis of ocular toxoplasmosis in blood, serum and tears in Brazil
Source: PLoS One. 2024 Feb 6;19(2):e0298393. doi: 10.1371/journal.pone.0298393 (PMC10846737; doi:10.1371/journal.pone.0298393)
Supplement: S1 Table — (DOCX) [file pone.0298393.s001.docx]

**Supplementary 1**

| **Gene** | **Primer** | **Cycling** | **Band size** | **Reference** |
| --- | --- | --- | --- | --- |
|  | **Primary PCR** | | | |
| B1 | JW63 GCACCTTTCGGACCTCAACAACCG JW62 TTCTCGCCTCATTTCTGGGTCTAC | 40 cycles Denaturation 1 min at 93°C Annealing 1 min at 55 °C Extension 2min 72°C Final extension 5 min 72°C | 286 bp | [22] |
|  | **Secondary PCR** | | | |
|  | B22 AACGGGCGAGTAGCACCTGAGGAGA  B23 TGGGTCTACGTCGATGGCATGACAACT | 25 cycles Denaturation 1 min at 94°C Annealing 30 seconds at 60°C Extension 1 min 72°C Final extension 10 min 72°C | 115 bp | [23] |
|  | **Primary PCR** | | | |
| REP 529 | Tox8 CCCAGCTGCGTCTGTCGGGAT Tox5 CGCTGCAGACACAGTGCATCTGGATT | 40 cycles Denaturation 1 min at 94°C Annealing 1 min at 55 °C Extension 2min 72°C Final extension 5 min 72°C | 450 bp | [24. 25] |
|  | **Secondary PCR** | | | |
|  | Tox9 AGGAGAGATATCAGGACTGTAC  ToxII GCGTCGTCTCGTCTAGATCG | 30 cycles Denaturation 30 seconds at 94°C Annealing 1min at 55°C Extension 1 min 72°C Final extension 5 min 72°C | 162 bp | [26. 27] |
|  | **Primary PCR** | | | |
| GRA7 | GRA7FE CAAGCACCCGTTGACAGTCT  GRA7RE ACGATGCACCCATACCAACAG | 30 cycles  Denaturation 40 seconds at 94°C  Annealing 40 seconds at 59 °C  Extension 40 seconds 63°C  Final extension 3 min 63°C | 322 bp | [28] |
|  | **Secondary PCR** | | | |
|  | GRA7FI CACCAGCATGGATAAGGCATC GRA7RI GCGAGCTTCTTCAGCAAGTCT | 30 cycles  Denaturation 40 seconds at 94°C  Annealing 40 seconds at 59 °C  Extension 40 seconds 63°C  Final extension 3 min 63°C | 222 bp | [28] |
