# Supplementary material for: Comparison of immunological and molecular methods for laboratory diagnosis of ocular toxoplasmosis in blood, serum and tears in Brazil
Source: PLoS One. 2024 Feb 6;19(2):e0298393. doi: 10.1371/journal.pone.0298393 (PMC10846737; doi:10.1371/journal.pone.0298393)
Supplement: S2 Table — *G1: Patient with active ocular toxoplasmosis; G2: patient with healed ocular toxoplasmosis; G3: patient with non-toxoplasmic uveitis; G4: patient without ocular injury. RE: right eye; LE: Left eye. F: Female sex; M: Male sex. DD: Disc Diameter. (DOCX) [file pone.0298393.s002.docx]

**Supplementary 2**

| **Information** | | **G1** | | **G2** | | **G3** | | **G4** | |
| --- | --- | --- | --- | --- | --- | --- | --- | --- | --- |
|  |  | **n** | **%** | **n** | **%** | **n** | **%** | **n** | **%** |
| **Sex** | F (=89) | 22 | 24.7% | 19 | 21.3% | 24 | 27.0% | 24 | 27.0% |
|  | M (=71) | 18 | 25.4% | 21 | 29.6% | 16 | 22.5% | 16 | 22.5% |
| **Consultation** | First consultation (=49) | 9 | 18.4% | 0 | 0.0% | 0 | 0.0% | 40 | 81.6% |
|  | More than one consultation (=111) | 31 | 27.9% | 40 | 36.0% | 40 | 36.0% | 0 | 0.0% |
| **Visual acuity** | RE - Normal vision (=75) | 9 | 12.0% | 19 | 25.3% | 19 | 25.3% | 28 | 37.3% |
|  | RE - Mild vision loss (=39) | 10 | 25.6% | 11 | 28.2% | 9 | 23.1% | 9 | 23.1% |
|  | RE - Moderate loss (=16) | 4 | 25.0% | 5 | 31.3% | 5 | 31.3% | 2 | 12.5% |
|  | RE - Severe loss (n=3) | 1 | 33.3% | 0 | 0.0% | 2 | 66.7% | 0 | 0.0% |
|  | RE - Deep loss up to the distance of 4 meters (n=13) | 7 | 53.8% | 4 | 30.8% | 2 | 15.4% | 0 | 0.0% |
|  | RE - Light perception (n=9) | 7 | 77.8% | 1 | 11.1% | 1 | 11.1% | 0 | 0.0% |
|  | RE - No light perception (n=5) | 2 | 40.0% | 0 | 0.0% | 2 | 40.0% | 1 | 20.0% |
|  | LE - Normal vision (n=80) | 16 | 20.0% | 21 | 26.3% | 13 | 16.3% | 30 | 37.5% |
|  | LE - Mild vision loss (n=42) | 11 | 26.2% | 11 | 26.2% | 13 | 31.0% | 7 | 16.7% |
|  | LE - Moderate loss (n=19) | 6 | 31.6% | 4 | 21.1% | 6 | 31.6% | 3 | 15.8% |
|  | LE - Severe loss (n=4) | 2 | 50.0% | 2 | 50.0% | 0 | 0.0% | 0 | 0.0% |
|  | LE - Deep loss up to the distance of 4 meters (n=5) | 2 | 40.0% | 1 | 20.0% | 2 | 40.0% | 0 | 0.0% |
|  | LE - Light perception (n=6) | 2 | 33.3% | 1 | 16.7% | 3 | 50.0% | 0 | 0.0% |
|  | LE - No light perception (n=4) | 1 | 25.0% | 0 | 0.0% | 3 | 75.0% | 0 | 0.0% |
| **Retinochoroiditis** | Unilateral (n=61) | 36 | 59.0% | 25 | 41.0% | 0 | 0.0% | 0 | 0.0% |
|  | Bilateral (n=19) | 4 | 21.1% | 15 | 78.9% | 0 | 0.0% | 0 | 0.0% |
|  | Absent (n=80) | 0 | 0.0% | 0 | 0.0% | 40 | 50.0% | 40 | 50.0% |
| **Eye injury site** | Central (n=37) | 17 | 45.9% | 20 | 54.1% | 0 | 0.0% | 0 | 0.0% |
|  | Periphery (n=37) | 23 | 62.2% | 14 | 37.8% | 0 | 0.0% | 0 | 0.0% |
|  | Central and periphery (n=6) | 0 | 0.0% | 6 | 100.0% | 0 | 0.0% | 0 | 0.0% |
|  | Absent (n=80) | 0 | 0.0% | 0 | 0.0% | 15 | 18.8% | 15 | 18.8% |
| **Number of eye injuries** | One (n=4) | 2 | 50.0% | 2 | 50.0% | 0 | 0.0% | 0 | 0.0% |
|  | Two (n=19) | 10 | 52.6% | 9 | 47.4% | 0 | 0.0% | 0 | 0.0% |
|  | More than two (n=56) | 27 | 48.2% | 29 | 51.8% | 0 | 0.0% | 0 | 0.0% |
| **Lesion Sizes** | Lesser than 1 DD (n=5) | 4 | 80.0% | 1 | 20.0% | 0 | 0.0% | 0 | 0.0% |
|  | Between 1 and 2 DD (n=56) | 30 | 53.6% | 26 | 46.4% | 0 | 0.0% | 0 | 0.0% |
|  | 3 DD (n=11) | 3 | 27.3% | 8 | 72.7% | 0 | 0.0% | 0 | 0.0% |
|  | 4 DD (n=6) | 1 | 16.7% | 5 | 83.3% | 0 | 0.0% | 0 | 0.0% |
